# Generation of cloned mice and nuclear transfer embryonic stem cell lines from urine-derived cells

**DOI:** 10.1038/srep23808

**Published:** 2016-04-01

**Authors:** Eiji Mizutani, Kohei Torikai, Sayaka Wakayama, Hiroaki Nagatomo, Yasuhide Ohinata, Satoshi Kishigami, Teruhiko Wakayama

**Affiliations:** 1 Faculty of Life and Environmental Sciences, University of Yamanashi, 4-4-37 Takeda, Kofu-shi, Yamanashi, Japan; 2 Advanced Biotechnology Center, University of Yamanashi, 4-4-37 Takeda, Kofu-shi, Yamanashi, Japan; 3 COC Promotion Center, University of Yamanashi, 4-4-37 Takeda, Kofu-shi, Yamanashi, Japan

## Abstract

Cloning animals by nuclear transfer provides the opportunity to preserve endangered mammalian species. However, there are risks associated with the collection of donor cells from the body such as accidental injury to or death of the animal. Here, we report the production of cloned mice from urine-derived cells collected noninvasively. Most of the urine-derived cells survived and were available as donors for nuclear transfer without any pretreatment. After nuclear transfer, 38–77% of the reconstructed embryos developed to the morula/blastocyst, in which the cell numbers in the inner cell mass and trophectoderm were similar to those of controls. Male and female cloned mice were delivered from cloned embryos transferred to recipient females, and these cloned animals grew to adulthood and delivered pups naturally when mated with each other. The results suggest that these cloned mice had normal fertility. In additional experiments, 26 nuclear transfer embryonic stem cell lines were established from 108 cloned blastocysts derived from four mouse strains including inbreds and F1 hybrids with relatively high success rates. Thus, cells derived from urine, which can be collected noninvasively, may be used in the rescue of endangered mammalian species by using nuclear transfer without causing injury to the animal.

Although the current success rate for producing live animals by cloning is low^1^, this technology has produced a variety of cloned animals for scientific and commercial purposes^2^. Cloned animals derived from somatic cells are almost identical to the original donor animals except for their mitochondrial DNA^3^. One interesting application of nuclear transfer (NT) techniques is the resurrection of extinct species and the rescue of endangered species.

It may be easier to rescue endangered species using NT techniques compared with resurrecting extinct species. However, in endangered species in existence at present, each individual is rare and precious, and it can be difficult to obtain donor cells and oocytes from these animals. Moreover, these endangered species are often protected by laws against hunting. Even for animals already in captivity, obtaining donor cells can confer a risk of injury or death. Recent studies have shown that oocytes and surrogate mothers might provide a substitute for a closely related “unendangered” species^4^,^5^, such as gaur bull cloning using domestic cows. By contrast, for donor cell collection, mice can be cloned from cells derived from one drop of blood^6^. Although this suggests that only a very small injury to the body (i.e., blood withdrawal) is needed to collect donor cells, there remains the risk of accidental death by injury caused by the need to restrain the animal for blood collection. Thus, it is preferable to find a way to collect donor cells noninvasively without causing any harm to the animal.

There are several methods to collect donor cells from animals noninvasively. For example, milk, especially colostrum, contains mammary gland epithelial cells, and cloned cows have been generated from these cell nuclei^7^. However, milk can be collected only from recently delivered females. By contrast, urine contains several types of somatic cells^8^, such as squamous epithelial cells from the urethra and bladder, and renal tubular cells^9^, and these cells can be cultured after collection^10^. Induced pluripotent stem (iPS) cells have been established from human urine-derived cells^11^,^12^, which suggests that urine-derived cells are a good candidate donor for NT. However, unlike domestic or zoo animals, there is a limited ability to collect urine-derived cells from wild animals and to collect the cells under clean conditions.

Cloned animals have been obtained from many different types of cells including mammary gland cells^13^, cumulus cells^14^, and fibroblasts^15^. However, it is not known whether urine-derived cells can be used for NT and whether healthy cloned animals can be generated from these cells. These cells spend a considerable amount of time stored in the high-osmolality and toxic urine environment until urination, and it is possible that this environment damages the donor nuclei. If cells contained in urine can be shown to be suitable as nuclear donors, they could provide donor cells for the generation of cloned animals without harming animals.

Here, we describe our studies to determine whether cells collected from mouse urine can provide donor nuclei to produce cloned mice without any treatment and to establish NT embryonic stem (ntES) cell lines.

## Results

### Collection of cells from urine

**Figure 1:**
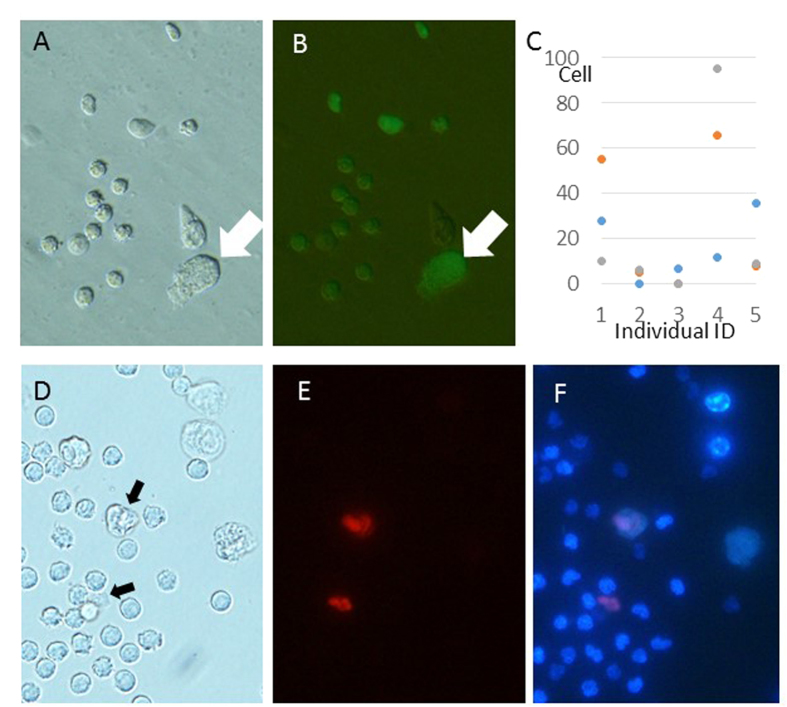
Mouse urine-derived cells. (**A,B**) Urine-derived cells derived from GFP-Tg mice. Those cells were collected from a drop of urine using a micromanipulator. Arrows show very large cells, which could not use for NT. (**C**) Urine-derived cell numbers for individual mice counted three times for each mouse. (**D–F**) Urine-derived cells were collected and stained with Hoechst stain and PI. Bright field (**D**), PI-positive cells (**E**), and Hoechst staining merged with PI staining. Arrows in (**D**) show dead cells, which can be distinguished without PI staining.

Observation of urine from green-fluorescent protein (GFP)-expressing transgenic (Tg) mice identified several types of cells. The large and keratinized cells could not be used as donors because they could not be injected into oocytes by hand (Fig. 1A,B, white arrows). The large but soft surface cells could be injected into enucleated oocytes using a very large injection pipette. However, none of the reconstructed oocytes could develop after activation (data not shown). The most frequent cell type was small and round, and had a clear surface. The number of these small cells varied between 0 and 95 cells per individual mouse (average 2–58 cells) (Fig. 1C). Staining of these cells with Hoechst stain and propidium iodide (PI) showed that almost any cell did not take up the PI dye, which suggested that most cells were viable. These cells were easy to distinguish from the PI-positive “dead” cells (Fig. 1D–F). We used the small viable cells for the following experiments.

### Development of reconstructed embryos

**Figure 2:**
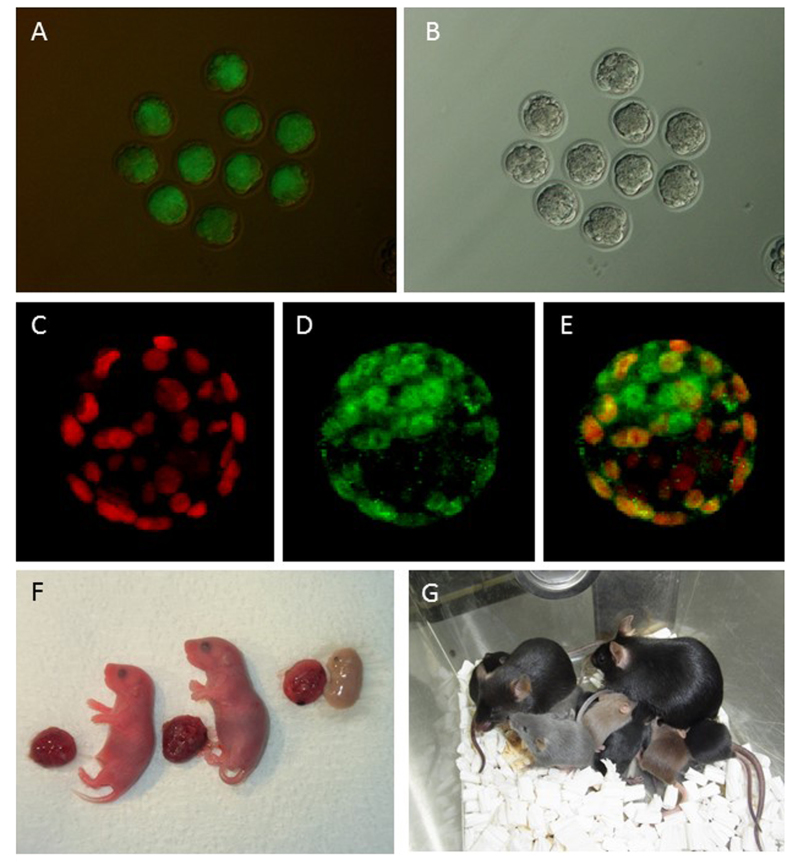
Cloned embryos and mice derived from urine-derived cell nuclei. (**A,B**) Cloned morulae/blastocysts derived from GFP-Tg mouse urine-derived cells. (**C–E**) Immunostaining of cloned blastocysts. Cdx2-positive cells are red (**C**), Oct4-positive cells are green (**D**) and their merged image is shown in (**E**). (**F**) Cloned offspring just after caesarean section. One dead foetus was also collected. (**G**) Cloned males and females derived from urine-derived cell nuclei were mated with each other. About 1 month later, female clones delivered their offspring, which suggested that the clones had normal fertility.

To examine the availability of urine-derived cells as nuclear donors, both sexes of several strains of mice were used. As shown in Table 1, after culturing cloned embryos for 72 h, about half of the cloned embryos reached the morula (46%) or blastocyst stage (2%) (Fig. 2A,B), which is similar to the rate for cumulus cell cloning^16^,^17^.

Immunostaining was used to evaluate the quality of blastocysts based on the cell number and allocation of inner cell mass (ICM) cells (Fig. 2C–E). The mean ICM and trophectoderm (TE) cell numbers were lower for BDF1 cloned blastocysts (ICM: 12.8, and TE: 32.5) than for fertilized embryos (ICM: 13, and TE: 68.8).

After embryo transfer at the two-cell stage, three female clones (one clone from a 129B6F1 mouse, aged 1 year, and two clones from BDF1 mice, aged 2–3 months), and one male clone (BDF1 mouse, aged 2–3 months) were generated form urine-derived cell nuclei (Table 2) (Fig. 2F). The success rate was 1–3%, which was slightly lower than the current success rate of cumulus cell cloning^18^. All cloned mice possessed a hypertrophic placenta, which is a typical abnormality in cloned mice^19^, but the body weights were within the normal range for cumulus cell-cloned mice^14^. One female clone died 2 weeks after birth, but all other mice grew to adulthood. When the male and female clones were mated, the females delivered the next generation (Fig. 2G), which suggested that these clones had normal fertility.

### Establishment of ntES cell lines

**Figure 3:**
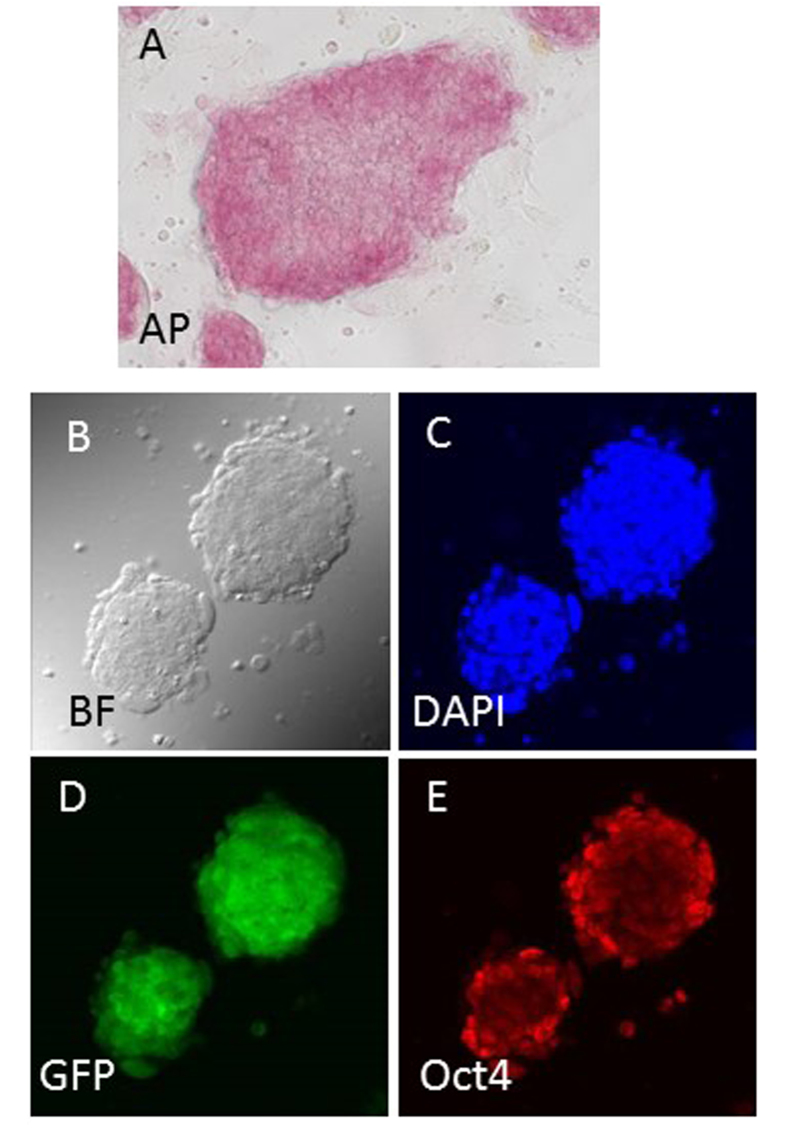
Immunostaining of ntES cell line from urine-derived cells. This ntES cell line was derived from 129GFP female mouse urine cell. AP staining (**A**), bright field (**B**), DAPI staining (**C**), GFP expression (**D**), and Oct4 immunostaining (**E**) of ntES cells. (**B–E)** are from the same sample.

We attempted to establish ntES cell lines from the cloned blastocysts derived from urine-derived cells. The establishment rate for ntES cell lines is much higher than that for the production of cloned mice^20^,^21^,^22^,^23^. Therefore, considering the current low success rate of animal cloning, we thought it better to establish ntES cell lines to secure the donor genome at the first attempt. In total, 26 ntES cell lines were established from 108 cloned blastocysts (24%) derived from urine-derived cells (Table 1). We selected six ntES cell lines randomly and applied alkaline phosphatase (AP) and immunofluorescent staining for the ES cell-specific undifferentiation markers Oct3/4. All cell lines were positive, as shown in Fig. 3. Although, the establishment rate differed between mouse strains (5–50%), at least one cell line could be established from each strain. The rates of establishment for ntES cell lines were 2–3 times greater than those for full-term clones (Tables 1 and 2).

Thus, these results suggest that urine-derived cells can be used as nuclear donors, as shown for other somatic cells, despite the exposure of these cells to urine in the body.

## Discussion

In this study, we demonstrate for the first time that mice can be cloned from intact urine-derived cells without harm to the animal’s body. There are several methods for collecting donor cells from animals noninvasively. For example, cells can be obtained from the skin surface by scratching or from hair follicles. However, these cells are usually dried and have a hardened cell surface compared with living cells. These cells can be used to extract DNA for the purpose of identifying victims or parentage. However, it is difficult to use these cells for NT for technical reasons.

In this study, two or three different types of cells could be identified in the mouse urine samples. These cells could be squamous epithelial cells from the urethra and bladder, and renal tubular cells^9^. Some could not be used because of their large size and hard cell surface. However, the small and round cells with a soft surface could be used as nuclear donors immediately after collection. It was easy to inject their nuclei into enucleated oocytes, and the ease of injection of these cells was similar or superior to the injection of cumulus cells and fibroblasts. Moreover, most of the urine-derived cells survived until use.

The ingredients contained in mammalian urine are not optimal for cell survival. The osmolality is high, and uric acid and ammonia are toxic. Although mice have the enzyme uricase^24^, which converts uric acid to the less toxic allantoin, urine is still not an appropriate environment for survival of somatic cells. Therefore, it was thought that even if live cells could be collected from urine^8^,^10^, the extreme environment would negatively affect cell survival and nuclear integrity. However, we generated male and female cloned mice from young and old mice, and demonstrated their fertility by mating them with each other and producing offspring. This strongly suggests that newly collected fresh and intact urine-derived cells are sufficiently viable to be used as nuclear donors for animal cloning.

In this study, we also tried to establish ntES cell lines from urine-derived cell nuclei. The establishment rate was high (24%) compared with full-term development (1–3%); these values are similar to those reported previously^22^,^23^. When the establishment rate was calculated for the urine-derived cells, one cell line was obtained from only 15 urine-derived cells (389 total NT/26 total established cell lines). Therefore, if few donor cells are available, it is better to establish ntES cell lines than to produce cloned mice. Once established, ntES cell lines will divide indefinitely or can be cryopreserved until use. Cloned animals can be produced from ntES cell nuclei via serial NT^20^,^21^,^25^,^26^. Thus, establishing ntES cell lines provides a better overall chance of cloning animals from endangered species. We are now trying to generate chimeric mice using these ntES cells.

The numbers and concentrations of cells in urine varied between individual mice, and some individuals provided only a few cells in urine (Fig. 1C). It is unclear why cell numbers differed between individual mice because all mice were housed in a clean facility (SPF condition) and therefore all could have been considered to be in good health. The number of cells collected for urine from wild animals is expected to vary widely between individuals and to reflect the state of health, such as the presence of urinary tract infection.

One advantage of the NT method is that only a few cells are required as nuclear donors. Although the current success rate of animal cloning is not high, cloning procedures and the success rates are continuing to improve with new methods such as adding histone deacetylase inhibitors to the medium^16^,^18^,^27^. The number of required donor cells will continue to decrease with increased success rate; therefore, in the future, fewer urine-derived cells will be needed to rescue endangered species.

Recently, one cloned cow was generated from urine-derived cells, but clean cells were selected and cultured until a sufficient number of cells was achieved after several passages^28^. However, in practical terms, it is difficult to collect fresh urine from individual animals in the field, especially from small animals. If urine is collected long after urination, the urine-derived cells may be infected or damaged, and it may be difficult to increase the number of cells by *in vitro* culture. Our previous study demonstrated that cloned mice could be produced even from dead cells of frozen cadavers^26^. Therefore, even if urine-derived cells are not fresh or are dead because of delayed collection, it may still be possible to produce cloned animals from endangered species. Further research is needed to determine how long urine-derived cells can survive after urination and whether dead cells can be used for NT.

In conclusion, our study suggests that urine may be a good source of cells for NT to rescue endangered species without causing harm to animals.

## Methods

### Animals

Urine-derived cells were collected from the following mice strains: B6D2F1 (C57BL/6 × DBA/2) male and female mice aged 8–10 weeks, 126B6F1 (129/Sv × C57BL/6) female mice aged about 1 year, C57BL/6 male and female mice aged 8–10 weeks, and pCX-eGFP 129/Sv Tg male and female mice aged 8–10 weeks. Oocytes were collected from B6D2F1 female mice aged 8–10 weeks. The surrogate pseudopregnant females used as embryo transfer recipients (see below) were ICR strain mice mated with vasectomized males of the same strain. B6D2F1, 129/Sv, C57BL/6, and ICR mice were purchased from Shizuoka Laboratory Animal Center (Hamamatsu, Japan). The 129B6F1 strain and Tg mice were bred in our mouse facility. All animal experiments conformed to the Guide for the Care and Use of Laboratory Animals and were approved by the Institutional Committee of Laboratory Animal Experimentation of the University of Yamanashi.

### Collection of oocytes

Mature oocytes were collected from the oviducts of 8–10-week-old female mice that had been induced to superovulate with 5 IU pregnant mare serum gonadotropin (Teikokuzoki, Tokyo, Japan) followed by 5 IU human chorionic gonadotropin (hCG, Teikokuzoki) 48 h later. Cumulus–oocyte complexes (COCs) were collected from the oviducts about 16 h after hCG injection. After collection, COCs were placed in HEPES-buffered CZB medium (H-CZB)^29^ and treated with 0.1% bovine testicular hyaluronidase (Sigma-Aldrich, St Louis, MO, USA). After several minutes, the cumulus-free oocytes were washed twice and then moved to a droplet of CZB medium for culture.

### Collection of urine-derived cells

**Figure 4:**
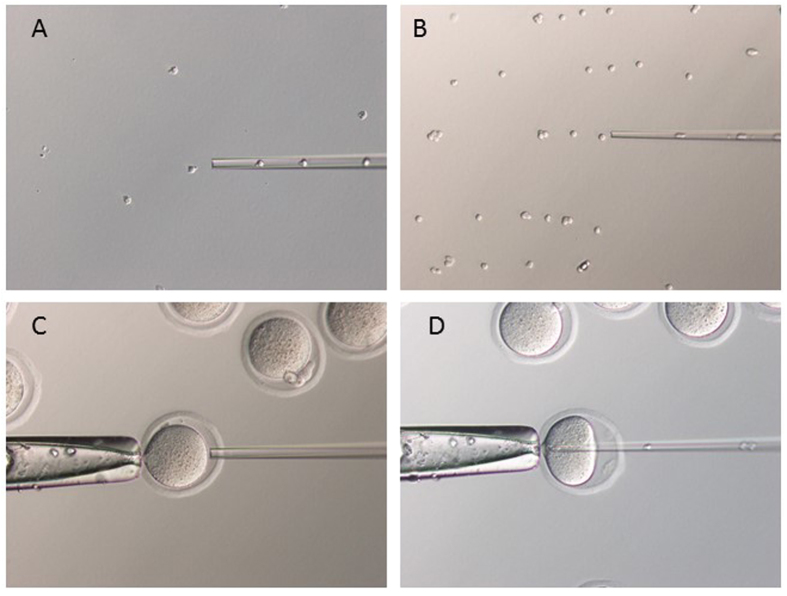
NT using urine-derived cells. (**A**) Cells were collected from urine with a micropipette and a micromanipulator. The inner diameter was about 10-15 μm. (**B**) The collected urine-derived cells were placed into PVP medium until use. (**C,D**) NT was performed using our standard method.

Randomly selected mice were caught by hand. Most of the mice discharged their urine immediately after being caught, and the urine was collected in 10 cm dishes. The volume of urine was measured with a pipette. The urine was placed in a dish covered with mineral oil, and the number of cells was counted for each mouse. In some cases, the urine-derived cells were collected by pipette (inner diameter: 10–15 μm) using a micromanipulator (Fig. 4A) and double stained with PI and Hoechst stain to measure the cell survival rate (Fig. 1). For NT, urine-derived cells were collected using a micromanipulator, placed individually and carefully into polyvinylpyrrolidone (PVP) medium, and kept until use (Fig. 4B).

### Nuclear transfer

The NT procedure was performed as described^14^,^30^. Groups of oocytes were transferred into a droplet of H-CZB containing 5 mg/ml cytochalasin B (CB) on the microscope stage for enucleation of the metaphase II (MII) spindle. Oocytes undergoing microsurgery were held with a holding pipette, and a hole was made in the zona pellucida using an enucleation pipette following the application of several piezo pulses (Prime Tech, Ibaraki, Japan). The MII chromosome–spindle complex was aspirated into the pipette with a minimum volume of ooplasm. After enucleation of all oocytes in one group, they were transferred into CZB. For nuclear injection, urine-derived cells and control cumulus cells were gently aspirated into and out of the injection pipette until their nuclei were largely devoid of visible cytoplasmic membrane. Each nucleus was injected immediately into an enucleated oocyte (Fig. 4C,D). The reconstructed oocytes were kept in an incubator until activation.

### Activation, culture and embryo transfer

The reconstructed oocytes were activated in 10 mM SrCl_2_ in Ca^2+^-free CZB medium in the presence of 50 nM trichostatin A (TSA) supplemented with 5 μM latrunculin A (Lat A) for 9 h^17^,^18^,^31^. Our original method for oocyte activation was 6 h with CB and TSA, but an additional 3 h culture was required without CB after washing of embryos. However, when Lat A was used instead of CB, oocytes could be cultured up to 9 h continuously. Formation on pseudopronuclei was examined, and the oocytes were cultured in CZB until embryo transfer at the two-cell stage. In additional experiments, some cloned embryos were cultured for 96 h to examine their potential for development to blastocysts or for 72 h to establish the ntES cell lines.

After the cloned embryos had developed to the two-cell stage the next day, they were transferred into the oviducts of pseudopregnant ICR strain female mice at 0.5 days post coitum (dpc) that had been mated with a vasectomized male the night before transfer. At 19.5 dpc, the offspring were delivered by caesarean section. After growing to adulthood, these mice were mated with other cloned mice as a measure of their fertility.

### Establishment of ntES cell lines

Cloned embryos were produced as described above and, when they reached the morula/blastocyst stage, were used to establish ntES cell lines as described^20^,^21^,^22^,^23^. The ICM was allowed to grow normally and, at passage 5–6, they formed as ES cell-like colonies. At this time, we considered these cells to be established and then randomly selected six cell lines for examination with AP staining and immunostaining to determine their pluripotency.

### Immunofluorescence and AP staining

The cell numbers in the blastocysts were estimated by counting the total number of nuclei and the number of TE nuclei stained with 4,6-diamidino-2-phenylindole (DAPI) and immunostaining for Cdx2, respectively. The cell number in the ICM was estimated as the total number of nuclei minus the number of TE nuclei. Immunofluorescent staining of cloned blastocyst and ntES cells was performed as described^32^,^33^,^34^. For immunostaining of the blastocysts, the primary antibodies used were an anti-Oct3/4 rabbit polyclonal antibody (1:200; Santa Cruz Biotechnology, Inc. Dallas, TX, USA) and anti-Cdx2 mouse monoclonal antibody (1:200; BioGenex, Inc., San Ramon, CA, USA) for detecting the ICM and TE. The secondary antibody was Alexa Fluor 488-labelled or 568-labelled goat anti-mouse IgG (Molecular Probes Inc., Eugene, OR, USA), respectively. DNA was stained with DAPI (2 mg/ml; Molecular Probes, Inc.). Established ES cell lines were tested for pluripotency with AP staining, according to the manufacturer’s protocol (Sigma-Aldrich). To test the normality of established ntES cell lines, the expression of Oct4 was examined using immunofluorescence staining with anti-Oct3/4 rabbit polyclonal antibody (1:200; Medical & Biological Laboratories Co., Ltd. Nagoya, Japan) and Alexa Fluor 568-labelled goat anti-rabbit IgG (Molecular Probes Inc., Eugene, OR, USA). In this study, two anti-Oct3/4 antibodies were used randomly because both worked well.

### Statistical analysis

Offspring development rates were evaluated using chi-squared tests. *P* < 0.01 was considered significant.

## Additional Information

**How to cite this article**: Mizutani, E. *et al*. Generation of cloned mice and nuclear transfer embryonic stem cell lines from urine-derived cells. *Sci. Rep*. **6**, 23808; doi: 10.1038/srep23808 (2016).

## Figures and Tables

**Table 1:** *In vitro* development and establishment of ntES cell lines from urine-derived cells.

Mouse strain	Sex	No. of oocytes used	Survived after activation	PN formation	Embryo development (72 h)				No. of plates	No. established (%)
					1–2 cells	4–8 cells	Morula (%)	Blastocyst (%)		
129B6F1	F	44	33	17	18	2	10 (59)	3 (18)	13	6 (46)^a^
B6D2F1	F	69	55	41	26	8	21 (51)	0	20	2 (10)^b^
129/Sv	F	57	47	42	19	12	16 (38)	0	16	4 (25)^c^
129/Sv	M	75	62	46	39	5	18 (39)	0	18	9 (50)^a,e^
C57BL/6	F	72	54	42	20	14	19 (45)	1 (2)	20	4 (20)^f^
C57BL/6	M	72	52	39	22	9	20 (51)	1 (3)	21	1 (5)^b,d^
Total		389	303	227	144	50	104 (46)	5 (2)	108*	26 (24)

PN: pronuclei

^a^vs^b^; ^c^vs^d^; ^e^vs^f^: *P* < 0.01.

*One cloned morula/blastocyst was lost before plating into medium.

**Table 2:** Production of cloned mice from urine-derived cells.

Mouse strain	Sex	No. of oocytes used	Survived after activation	PN formation	Embryo development		No. of ETs	No. of cloned mice (%)*	Weight at birth (g)	
					Frag. or 1-cell	2-cell (%)			Body	Placenta
129B6F1	F	105	86	72	26	60 (83)	60	1 (2)	1.83	0.50
BDF1	F	261	221	202	66	155 (77)	155	2 (1)	2.20, 1.61	0.26, 0.28
BDF1	M	96	87	37	55	32 (86)	32	1 (3)	1.82	0.27

PN: pronuclei; Frag; fragment; ETs: embryo transfers.

^*^There were no significant differences between mouse strains.
